# Extragenital Infections Caused by* Chlamydia trachomatis* and* Neisseria gonorrhoeae*: A Review of the Literature

**DOI:** 10.1155/2016/5758387

**Published:** 2016-06-05

**Authors:** Philip A. Chan, Ashley Robinette, Madeline Montgomery, Alexi Almonte, Susan Cu-Uvin, John R. Lonks, Kimberle C. Chapin, Erna M. Kojic, Erica J. Hardy

**Affiliations:** ^1^Division of Infectious Diseases, Department of Medicine, Warren Alpert Medical School, Brown University, Providence, RI 02903, USA; ^2^Department of Pathology, Rhode Island Hospital, Providence, RI 02903, USA

## Abstract

In the United States, sexually transmitted diseases due to* Chlamydia trachomatis* and* Neisseria gonorrhoeae* continue to be a major public health burden. Screening of extragenital sites including the oropharynx and rectum is an emerging practice based on recent studies highlighting the prevalence of infection at these sites. We reviewed studies reporting the prevalence of extragenital infections in women, men who have sex with men (MSM), and men who have sex only with women (MSW), including distribution by anatomical site. Among women, prevalence was found to be 0.6–35.8% for rectal gonorrhea (median reported prevalence 1.9%), 0–29.6% for pharyngeal gonorrhea (median 2.1%), 2.0–77.3% for rectal chlamydia (median 8.7%), and 0.2–3.2% for pharyngeal chlamydia (median 1.7%). Among MSM, prevalence was found to be 0.2–24.0% for rectal gonorrhea (median 5.9%), 0.5–16.5% for pharyngeal gonorrhea (median 4.6%), 2.1–23.0% for rectal chlamydia (median 8.9%), and 0–3.6% for pharyngeal chlamydia (median 1.7%). Among MSW, the prevalence was found to be 0–5.7% for rectal gonorrhea (median 3.4%), 0.4–15.5% for pharyngeal gonorrhea (median 2.2%), 0–11.8% for rectal chlamydia (median 7.7%), and 0–22.0% for pharyngeal chlamydia (median 1.6%). Extragenital infections are often asymptomatic and found in the absence of reported risk behaviors, such as receptive anal and oral intercourse. We discuss current clinical recommendations and future directions for research.

## 1. Introduction

Sexually transmitted diseases (STDs) continue to be a significant cause of morbidity in the United States (US) with an estimated $15.9 billion spent annually on healthcare costs related to their diagnosis and treatment [1]. The two most common reportable bacterial STDs in the US are gonorrhea and chlamydia [2]. Chlamydia is caused by the bacterium* Chlamydia trachomatis* and is the most commonly reported STD. In 2014, over 1.4 million cases of chlamydia were diagnosed in the US [2], a 2.8% increase from the prior year and the greatest number of cases ever reported for an STD. Of chlamydia cases in 2014, the majority were among younger adults age 15–24 and women (70%). Despite overall higher prevalence of chlamydia infection among women in the US, diagnoses among men increased by 6.8% from 2013 to 2014. The difference in chlamydia diagnoses by gender can likely be attributed to routine screening practices among women [2]. The major primary care guidelines in the US recommend annual chlamydia screening of all sexually active young women (age 24 years and younger) as part of annual routine reproductive healthcare services [3]. Similar to chlamydia, gonorrhea also disproportionately impacts younger populations. Gonorrhea is caused by the bacterium* Neisseria gonorrhoeae* with over 350,000 cases reported in 2014, a 5.1% increase from the prior year and a 10.5% increase since 2010 [2]. Unlike chlamydia, gonorrhea is now more prevalent among men than women. The number of gonorrhea cases among men increased by 27.9% from 2010 to 2014, whereas the number of cases among women decreased by 4.1% during that time. The rising number of new chlamydia and gonorrhea cases among men is likely due to increased diagnoses among gay, bisexual, and other men who have sex with men (MSM) [4, 5].

Gonorrhea and chlamydia are often asymptomatic in men as well as women. In men, only 14% infected with chlamydia and 40% infected with gonorrhea may be symptomatic [6, 7]. In women, urogenital chlamydia initially infects the cervix, causing symptoms of cervicitis which can then spread to the upper reproductive tract and cause pelvic inflammatory disease (PID). Untreated urogenital infections can lead to other serious complications such as chronic pain, ectopic pregnancy, and infertility [8]. The presence of gonorrhea or chlamydia at any site also increases the risk of acquiring HIV in both men and women [9, 10]. Complications specific to men include epididymitis, prostatitis, and proctitis. Both men and women with symptomatic urogenital infection most commonly present with urethritis, characterized by dysuria and urethral discharge. Reactive arthritis may also occur, often as part of a triad of other symptoms including urethritis and conjunctivitis [11].

*N. gonorrhoeae* and* C. trachomatis* can also be detected in the pharynx and rectum [2]. Gonorrhea and chlamydia infection in the rectum can cause rectal pain, bleeding, and discharge, as well as proctitis. In the pharynx, these infections can cause symptoms, such as pharyngitis and lymphadenitis, but are most often asymptomatic. Given that extragenital testing is not always part of routine STD screening, particularly in the absence of symptoms, many extragenital infections are undiagnosed and untreated. These untreated extragenital infections are a potential reservoir for ongoing transmission and may also lead to increased risk of HIV acquisition. Extragenital testing for* N. gonorrhoeae* and* C. trachomatis* is an emerging area that should be considered in both men and women. We review current screening recommendations and evidence to support extragenital testing for* N. gonorrhoeae* and* C. trachomatis* and discuss areas where future research is needed.

## 2. Materials and Methods

Current guidelines related to extragenital screening for* N. gonorrhoeae* and* C. trachomatis* in men and women were reviewed. A literature review was performed of all studies listed in PubMed evaluating extragenital gonorrhea and chlamydia infections through December 1, 2015. Studies included those describing extragenital infections by* N. gonorrhoeae*,* C. trachomatis*, or both, conducted in the US as well as internationally. The goal of the review was to describe the current epidemiology and prevalence of extragenital infections in the setting of the latest recommendations for screening. We specifically examined extragenital infections in separate subgroups of populations, including women, men who have sex only with women (MSW), and MSM. Only studies in English were included. The search terms “extragenital,” “rectal,” “pharyngeal,” “chlamydia,” and “gonorrhea” were used in combination and individually. References were reviewed and subsequently excluded if the study did not include findings of extragenital* N. gonorrhoeae* or* C. trachomatis* infection. Additionally, citations within these studies were reviewed and included if relevant. Full texts of relevant studies were retrieved and reviewed.

## 3. Results and Discussion

### 3.1. Current Screening Recommendations

The Centers for Disease Control and Prevention (CDC) currently recommends that all sexually active women less than 25 years of age, as well as older women who have specific risk factors (e.g., new or concurrent sex partners), be tested annually for urogenital chlamydia and gonorrhea infection [12]. Per the guidelines, the clinical significance of pharyngeal chlamydia infection is unclear and routine pharyngeal screening for chlamydia is not recommended [12–14]. The US Preventive Services Task Force (USPSTF), the preeminent primary care guidelines in the US, recommends screening for chlamydia and gonorrhea in all sexually active women of age 24 years and younger, and in older women who are at increased risk for infection (e.g., due to another current STD, a previous STD, new or concurrent sex partners, inconsistent condom use, drug use, commercial sex work, certain demographic characteristics, or high community prevalence of STDs). The American Congress of Obstetricians and Gynecologists (ACOG) recommends annual urogenital screening for gonorrhea and chlamydia for sexually active women age 25 years and younger, as well as for women over age 25 reporting risk factors for infection [15].

The CDC does not recommend routine chlamydia or gonorrhea screening in men [12], with the exception of “considering” screening in high-prevalence clinical settings such as STD clinics or among high-prevalence populations such as MSM. The CDC recommends that MSM be screened at least annually for chlamydia infection at sites of sexual contact, including the rectum and urethra; for gonorrhea, the guidelines recommend screening at the urethra, rectum, and pharynx. Per these guidelines, screening should be based on risk behaviors. MSM who report insertive sex should be screened for urogenital* N. gonorrhoeae* and* C. trachomatis*. MSM who report receptive anal sex should be screen for rectal* N. gonorrhoeae* and* C. trachomatis*. MSM who report receptive oral sex should be screened for pharyngeal* N. gonorrhoeae* only; screening for* C. trachomatis* pharyngeal infection is not recommended. The USPTF does not recommend screening for chlamydia or gonorrhea in MSW due to insufficient evidence to support this practice.

The majority of international STD treatment guidelines provide recommendations for extragenital testing in MSM. The International Union Against Sexually Transmitted Infections (IUSTI) recommends extragenital testing for both MSM and women at the rectum and pharynx if there is a reported history of sexual exposure [16, 17]. Similarly, the British Association for Sexual Health and HIV (BASHH) recommends that extragenital screening for chlamydia and gonorrhea infections be dependent on reported sexual behaviors among men and women [18]. The guidelines also recommend extragenital testing among specific groups of women, such as commercial sex workers [19, 20]. Other countries, such as South Africa, employ an algorithm-driven, syndromic approach to STD testing and treatment [21].

The group of women who have sex with women (WSW) encompasses a diverse set of individuals and sexual practices. The CDC addresses this unique group, recommending that screening for* N. gonorrhoeae* and* C. trachomatis* be based on a detailed history of sexual practices [12]. The CDC also specifically addresses transgender men and women and recommends STD risk assessment and testing be based on current anatomy and sexual behaviors in this group [12].

### 3.2. Overview of Existing Literature

A total of 80 studies were reviewed focusing on extragenital infection with* N. gonorrhoeae* or* C. trachomatis*. Studies were published between 1981 and 2015 and included sites in North America (*n* = 37), Europe (*n* = 29), Australia (*n* = 9), Asia (*n* = 4), and Africa (*n* = 2). Study settings included STD clinics (*n* = 38), other outpatient clinics (*n* = 10), genitourinary clinics (*n* = 7), HIV clinics (*n* = 9), gay men's health centers (*n* = 3), community-based and outreach settings (*n* = 6), and other settings (*n* = 3); a minority of studies presented findings from multiple sites (*n* = 6). Most studies evaluated a single population but some did include multiple populations of women, MSW, and MSM. The number of studies reporting specific populations included MSM (*n* = 54), women (*n* = 33), MSW (*n* = 9), and mixed populations (*n* = 9). The following sections describe extragenital* N. gonorrhoeae* and* C. trachomatis* infection in these different populations.

### 3.3. Extragenital Infections in Women

A total of 33 studies reported prevalence of extragenital infection in women due to* N. gonorrhoeae* or* C. trachomatis* infection [19, 22–52] (Table 1). The range of prevalence of extragenital infections reported was 0.6–35.8% for rectal gonorrhea (median 1.9%), 0–29.6% for pharyngeal gonorrhea (median 2.1%), 2.0–77.3% for rectal chlamydia (median 8.7%), and 0.2–3.2% for pharyngeal chlamydia (median 1.7%). Most study sites were STD clinics and other high-risk settings; few were primary care settings, clinics focusing on women's care, or centers focusing on transgender patient care.

Most extragenital infections in women are asymptomatic, with estimates including 93% of pharyngeal gonorrhea [39], 53–100% of rectal gonorrhea [39], 100% of pharyngeal chlamydia [39], and 36–100% of rectal chlamydia cases [29, 30, 39]. Furthermore, a significant number of women who test positive for rectal gonorrhea or chlamydia do not report anal sex [19, 29, 53]. Extragenital screening increases the yield of detection of either gonorrhea or chlamydia at pharyngeal or rectal sites by approximately 6–50% or greater in women compared to screening urogenital specimens alone [23–25, 27, 29–31, 39, 44–46]. Overall, reported risk factors for rectal infection in women include younger age (*n* = 2 studies), sex with an injection drug user (*n* = 1), exchanging sex for money (*n* = 2), anonymous partners (*n* = 1), a sex partner with gonorrhea or chlamydia (*n* = 1), and sex while under the influence of drugs or alcohol (*n* = 1) [23, 31, 48]. However, other studies have not found any associations with these risk factors [29, 30].

Based on prevalence data, universal screening for extragenital infection due to* N. gonorrhoeae* or* C. trachomatis* in settings which care for women who are at risk of these infections (e.g., those who are sexually active with concurrent or nonmutually monogamous partners, regardless of reported exposure sites) should be considered. Due to the frequency of asymptomatic extragenital infections and the inaccuracy of testing based on self-reported behavior [19], the evidence supports routine screening in high-risk settings such as STD clinics. Universal screening for extragenital infection will certainly increase case finding, which in turn will likely have both clinical and public health benefits such as avoiding reproductive health sequelae and limiting HIV transmission. However, extragenital screening protocols among sexually active women are not currently widespread, and further study is needed to evaluate the impact on sexual health outcomes. Additionally, given the paucity of extragenital infection studies in other settings (e.g., primary care clinics), prevalence in these settings is largely unknown and merits further study.

### 3.4. Extragenital Infections in MSM

A total of 53 studies evaluated the prevalence of extragenital infections due to* N. gonorrhoeae* or* C. trachomatis* in MSM [13, 19, 22, 25–27, 36, 38, 44, 45, 47–49, 54–93] (Table 2). Extragenital infections among MSM have been studied more extensively compared to women. MSM experience high rates of both extragenital gonorrhea and chlamydia. The prevalence of extragenital infection among MSM in these studies ranged from 0.2–24% for rectal gonorrhea (median 5.9%), 0.5–16.5% for pharyngeal gonorrhea (median 4.6%), 2.1–23% for rectal chlamydia (median 8.9%), and 0–3.6% for pharyngeal chlamydia (median 1.7%); the differences are due to different clinical settings and methods of diagnosis. Several studies have evaluated the national prevalence of extragenital infections among MSM in the US [12, 24, 29, 30, 35, 43, 59, 63–65, 69–71, 73, 74, 76–78, 80, 82, 83]. In a large cohort of 3,034 MSM who attended a STD clinic in Seattle, Washington in 2011, extragenital infections were common and included pharyngeal gonorrhea (6.5%) and chlamydia (2.3%), and rectal gonorrhea (9.7%) and chlamydia (11.9%) [57]. Fifty-seven percent of cases were found in only extragenital sites (nonurogenital).

Similarly, among 21,994 MSM screened as part of the CDC STD Surveillance Network, composed of 42 STD clinics across the US, the prevalence of infection was 7.9% for pharyngeal gonorrhea, 2.9% for pharyngeal chlamydia, 10.2% for rectal gonorrhea, and 14.1% for rectal chlamydia. Over 70% of extragenital infections in this sample would have been missed with urogenital screening alone. In summary, urogenital testing alone misses a significant percentage of gonorrhea and chlamydia infections among MSM; if MSM were screened for urogenital infections alone, 14% to 85% of rectal and oropharyngeal gonorrhea and chlamydia infections would have been missed [22, 57, 63, 64, 68, 79, 80].

The majority of extragenital infections among MSM are asymptomatic, with estimates ranging from 25% to 100% from reported studies [68, 76, 80, 89, 92, 94]. Men with extragenital gonorrhea may be more likely to be symptomatic than those with chlamydia [25, 62, 71, 95, 96]. For example, in one large study of MSM with extragenital infection, only 5.1% of pharyngeal and 11.9% of rectal infections were symptomatic with the most common pharyngeal symptoms being pharyngitis (65%), localized lymphadenopathy (16%), and inflammation of the oral cavity (10%). The most common rectal symptoms were pruritus (36%) anal discharge (17%), burning (13%), inflammation (11%), pain (11%), and erythema around the anus (6%) [62]. Symptom-based screening may miss up to 60% of extragenital infections [30, 39, 45].

Extragenital infections may also be increasing in prevalence [59, 77, 95], as several studies have reported higher prevalence of extragenital infections among MSM in recent time periods. However, this could also reflect more thorough screening practices or improved testing methods [97, 98]. Extragenital infections among MSM are associated with concurrent partners, existing HIV infection (*n* = 2 studies), condomless anal sex (*n* = 3), and drug use during sex (*n* = 1) [62, 65, 69, 89, 91]. Concurrent infections with other STDs are common [99]. The overwhelming evidence indicates a high prevalence of extragenital* N. gonorrhoeae* and* C. trachomatis* infections among MSM, the asymptomatic nature of most of these infections, and the prevalence of extragenital infection without concurrent urogenital infection, all of which support the need for routine screening at extragenital sites.

### 3.5. Extragenital Infections in MSW

A total of nine studies evaluated the prevalence of extragenital infections due to* N. gonorrhoeae* or* C. trachomatis* in MSW [19, 26, 27, 33, 36, 38, 44, 45, 100] (Table 3). Overall, there are limited prevalence data of extragenital infections among MSW. The prevalence of extragenital infections among MSW in the studies reviewed ranged 0–5.7% for rectal gonorrhea (median 3.4%), 0.4–15.5% for pharyngeal gonorrhea (median 2.2%), 0–11.8% for rectal chlamydia (median 7.7%), and 0–22.0% for pharyngeal chlamydia (median 1.6%). These data represent studies that evaluated heterosexually identified men, some of whom may have engaged in sex with other men [44], a distinction which emphasizes the need to consistently focus on sexual behavior rather than identity. Other studies which did not evaluate for or stratify by specific risk behaviors further support the prevalence studies in individual populations [32, 100–107] (Table 3).

### 3.6. Diagnoses of Extragenital Infections

The gold standard for diagnosis of urogenital infection due to* N. gonorrhoeae* and* C. trachomatis* is the nucleic acid amplification test (NAAT). However, NAAT assays are not approved by the US Food and Drug Administration (FDA) for detecting* N. gonorrhoeae* and* C. trachomatis* from pharyngeal or rectal specimens [108]. Culture is still the only approved method for diagnosis at these sites. However, NAAT is the most sensitive test for detecting* C. trachomatis* and* N. gonorrhoeae* and is recommended for this purpose by the CDC [12]. NAAT has demonstrated higher sensitivity and specificity compared to culture for detecting extragenital infections [52, 74, 109, 110]. At the present time, laboratories must validate these tests in-house based on Clinical Laboratory Improvement Amendments (CLIA) regulatory requirements before performing NAAT testing on rectal and pharyngeal specimens; many large commercial laboratories have performed this validation and offer this testing option. The main disadvantages of performing NAAT testing over culture is the inability to determine antimicrobial susceptibilities and bacterial viability. Potentially lower sensitivity of NAAT for* N. gonorrhoeae* in the pharynx and rectum may be linked to substantial colonization of these extragenital sites by a wide range of other organisms, including other Neisseria species, possibly leading to interference with* N. gonorrhoeae* isolation [111]. For suspected or documented treatment failure,* N. gonorrhoeae* cultures should be obtained and antimicrobial susceptibilities performed.

Extragenital specimens are collected via a swab of the rectum or pharynx, by either a clinician or a self-collected swab. Self-collected swab as a means of collecting pharyngeal and rectal specimens is supported by the CDC guidelines [12] and has been found to be an acceptable means of obtaining specimens among women [112, 113] and MSM [49, 54, 85, 114–117], which may lead to an increase in extragenital diagnoses [118] due to the noninvasive nature of the procedure. Self-collected swabs may also reduce the workload for clinic staff who obtain them and promote screening when clinicians are not available for collection.

### 3.7. Treatment of Extragenital Infections

Current US guidelines regarding treatment of extragenital infections due to* N. gonorrhoeae* and* C. trachomatis* are similar to those for the treatment of urogenital infections [12]. Treatment guidelines from the United Kingdom and Europe both recommend similar regimens for both urogenital and extragenital infections [17, 18, 119]. Extragenital pharyngeal and rectal gonorrhea and chlamydia infections may spontaneously clear even in the absence of treatment among MSM and high-risk women [120, 121]. If extragenital sites are a reservoir for ongoing transmission, then suboptimal treatment of extragenital infections could lead to the spread of any existing resistant organisms. Care should be taken with extragenital treatment and retesting should be performed if persistent infection or treatment failure is suspected.

The recommended treatment for urogenital chlamydia infection is azithromycin 1000 milligrams orally in a single dose or doxycycline 100 milligrams orally twice daily for seven days. Due to the ease of administration, the ability for directly observed therapy in a single dose, and the high rates of adherence, azithromycin is the usual treatment option in many clinics. Earlier reports demonstrated similar results with both regimens for treatment of urogenital infection, with high (>96%) cure rates [122]. In contrast, recent analyses have suggested a potential small advantage of doxycycline compared to azithromycin for urogenital chlamydia infection; the efficacy of doxycycline has been reported as being 100% compared to 97% for azithromycin [123, 124].

Efficacy of chlamydia treatments may differ for extragenital infections at rectal and pharyngeal sites [19]. Doxycycline may have slightly greater efficacy compared to azithromycin for both rectal [125–132] and pharyngeal [133] chlamydia infection, as single-dose azithromycin may not lead to sustained drug concentrations capable of curing extragenital infection [134]. For example, treatment failure was significantly more common with azithromycin (10% of patients) compared to doxycycline (2%) in a small study for treatment of pharyngeal chlamydia. In a meta-analysis of azithromycin and doxycycline for the treatment of rectal chlamydia, azithromycin was 83% effective compared to >99% efficacy of doxycycline [125]. Treatment guidelines in Europe [16] and Australia [135] recommend doxycycline as the treatment of choice for rectal infections. However, care should be taken when interpreting these smaller studies, and the potential small benefits must be weighed against the ease of administration, ability for directly observed therapy, and adherence for the single dose azithromycin therapy option. No randomized controlled trials have evaluated treatment regimens for extragenital chlamydia infection and further studies are needed to determine optimal management of these infections [136].

The current treatment recommendations for urogenital* N. gonorrhoeae* infections involve a dual regimen of ceftriaxone 250 milligrams intramuscularly as a single dose in addition to azithromycin 1000 milligrams orally in a single dose [12]. Uncomplicated rectal infections with* N. gonorrhoeae* should be treated in the same manner. Given that both ceftriaxone and azithromycin are administered as a single dose, these drugs should be administered together and under direct observation. These recommendations are based on a number of treatment failures with ceftriaxone alone and an increasing minimum inhibitory concentration (MIC) to oral cephalosporins which has been observed mostly outside of the US [137–148]. The dual therapy also has the advantage of treating* C. trachomatis* infection, which frequently accompanies* N. gonorrhoeae* infection. Doxycycline can be considered in place of azithromycin, but azithromycin is strongly preferred given increased resistance to doxycycline [144]. This regimen has a high (>98%) treatment efficacy for rectal infections [149, 150]. Pharyngeal infections with* N. gonorrhoeae* are more difficult to treat and have demonstrated ceftriaxone resistance and treatment failure in a number of countries outside the US [138–141, 143, 145, 146, 151, 152]. In both pharyngeal and rectal gonorrhea, persistence of the organism after treatment may be due to reinfection but can also reflect an elevated MIC to antibiotic regimens [153]. At this time, guidelines still recommend treating pharyngeal infection by* N. gonorrhoeae* with ceftriaxone and azithromycin [12]. The addition of azithromycin may improve treatment efficacy for pharyngeal infections [154, 155].

In general, test of cure is not recommended except in cases where there are persistent symptoms, therapy was not completed, or reinfection is suspected. Retesting for both urogenital and extragenital infections less than three weeks after treatment is not recommended and can result in false positive results due to the highly sensitive nature of NAAT and the possibility of detection of nonviable organisms [156, 157]. Furthermore, due to NAATs not being FDA-cleared at this time for the purpose of testing for cure, culture is the only retesting method that can be used to properly assess the efficacy of antibiotic treatments. The significance of positive NAAT at extragenital sites during this time is unclear and should be interpreted after a detailed clinical interview including presence or absence of symptoms, potential risk for reinfection, and adherence to treatment [158]. Men and women who are positive for* N. gonorrhoeae* and* C. trachomatis* should generally be tested for reinfection three to six months after treatment [12, 16].

## 4. Conclusions

Several key questions exist regarding screening for and management of extragenital infections. Urogenital screening for* N. gonorrhoeae* and* C. trachomatis* infection is generally performed to reduce complications in women and to decrease the risk of HIV infection in MSM [159–162]. However, there is a lack of data on clinical outcomes associated with rectal and pharyngeal infections, including impact on overall morbidity. Two major questions are whether routine screening and treatment for extragenital gonorrhea and chlamydia infections in women prevent sequelae observed in urogenital infection (such as PID, ectopic pregnancy, and infertility), and whether routine screening and treatment reduces the risk of HIV transmission in MSM. With regard to management, optimal treatment regimens for rectal and pharyngeal extragenital infections is unknown. Asymptomatic extragenital infections may be a reservoir of ongoing transmission and antibiotic resistant strains from these reservoir sites may go undetected and promote the spread of resistance.

The contribution of extragenital infections to overall transmission of gonorrhea and chlamydia, including the transmission potential between different anatomic sites, is also unclear. In women, evidence suggests that rectal infections can be spread to urogenital sites [163]. It is also likely that pharyngeal infections can be spread to the male urethra [13, 14, 164] and rectum [165]. Contributing to potential transmission risk may be bacterial load at different anatomic sites [166, 167]. These data suggest that the prevalence and associated morbidity of extragenital infections caused by* N. gonorrhoeae* and* C. trachomatis*, especially among women, may be reduced by thorough extragenital screening and early treatment of extragenital infections, although this is unproven. Screening and treatment for rectal infections, especially among populations at high risk of HIV (e.g., MSM), may be a cost-effective intervention to prevent HIV [168]. Optimal screening strategies for extragenital infections are largely unknown. Further studies are needed in settings other than reproductive health and STD clinics, especially in primary care clinics and resource-limited settings.

Extragenital infections due to* N. gonorrhoeae* and* C. trachomatis* are common, especially in settings which provide services to higher-risk men and women. In general, MSM demonstrate a higher prevalence of extragenital infection compared to women and MSW [22, 25–27, 44, 47–49]. Despite the accumulating data on the prevalence of these infections, screening at extragenital sites remains uncommon [101, 102, 169]. STD and other sexual health clinics should consider implementing routine, universal extragenital screening for* N. gonorrhoeae* and* C. trachomatis* infection among high-risk men and women. Importantly, guidelines suggest screening based on reported risk behaviors; however, this may miss a significant amount of extragenital infection [19, 22, 45, 47, 49, 52, 59, 62, 68, 77–79, 90, 103, 107, 120, 133, 170, 171]. In addition to targeting those with symptoms and those reporting condomless anal or oral sex, screening should also include those without symptoms and those who do not report condomless sex at a specific extragenital site, as the nature of the infections are often asymptomatic, and high-risk behaviors are not consistently reported by patients.

## Figures and Tables

**Table 1:** Characteristics of studies reporting extragenital infections due to *N. gonorrhoeae *and* C. trachomatis* among women.

Author	Study year	Location	Setting	Time period	Risk	Sample	Gonorrhea			Chlamydia		
							Urogenital	Rectal	Pharyngeal	Urogenital	Rectal	Pharyngeal
Jones et al. [33]	1985	Indiana (US)	STD clinic	1985	Women	686/1,223	—	—	—	28.00%	5.20%	3.20%
Ostergaard et al. [38]	1997	Denmark	STD clinic	1995-1996	Women	196	—	—	—	3.60%	3.10%	1.50%
Linhart et al. [35]	2008	Tel Aviv, Israel	Sex worker outreach	2008	Women	300	5.00%	—	9.00%	6.30%	—	—
van der Helm et al. [49]	2009	Netherlands	STD clinic	2006-2007	Women	936	—	2.00%	—	—	9.00%	—
Barry et al. [24]	2010	California (US)	STD clinic	2007-2008	Women	1,308	1.80%	1.70%	—	5.40%	4.70%	—
Giannini et al. [28]	2010	Ohio (US)	STD clinic, children's hospital	2003–2007	Women	1,949	5.6–9.3%	2.9–13.4%	2.5–6.8%	—	—	—
Hunte et al. [30]	2010	Florida (US)	STD clinic	2007	Women	97	8.20%	13.40%	—	16.50%	17.50%	—
Raychaudhuri and Birley [41]	2010	United Kingdom	Genitourinary clinic	2005–2008	Women	120	27.50%	35.80%	—	—	—	—
Sethupathi et al. [42]	2010	United Kingdom	Genitourinary clinic	2006–2008	Women	152	—	—	—	11.90%	12.50%	—
Peters et al. [39]	2011	Netherlands	STD clinic	2007-2008	Women	4,299	1.10%	1.70%	0.80%	10.00%	8.70%	1.90%
Javanbakht et al. [31]	2012	California (US)	STD clinic	2008–2010	Women	2,084	3.30%	3.00%	—	12.00%	14.60%	—
Koedijk et al. [22]	2012	Netherlands	STD clinic	2006–2010	Women	207,134	1.00%	1.20%	1.20%	10.40%	9.30%	2.70%
Mayer et al. [36]	2012	US (4 cities)	HIV clinic	2004–2006	Women (HIV+)	119	1.00%	1.00%	2.00%	3.00%	2.00%	2.00%
Diaz et al. [26]	2013	Spain	STD clinic	2006–2010	Women	318	0.90%	3.10%	29.60%	—	—	—
Shaw et al. [43]	2013	United Kingdom	Genitourinary clinic	2013	Women	2,808	0.50%	0.60%	0.30%	6.70%	7.10%	1.30%
van Liere et al. [45]	2013	Netherlands	STD clinic	2010-2011	Women	1,321	1.30%	0.90%	2.30%	5.40%	4.80%	1.40%
Dimech et al. [102]	2014	Australia	Surveillance system	2008–2010	Women	415,069	—	—	—	6.7—10%	5.20%	1.70%
Jenkins et al. [32]	2014	Illinois (US)	Emergency room	2012-2013	Women	301	1.00%	—	0.70%	6.30%	—	0.70%
Ladd et al. [34]	2014	US (multiple cities)	Mail	2009–2011	Women	205	—	2.50%	—	—	11.10%	—
Peters et al. [40]	2014	South Africa	Primary care clinic	2011-2012	Women	604	10.00%	2.50%	0.00%	16.00%	7.10%	0.20%
van Liere et al. [46]	2014	Netherlands	STD clinic	2012-2013	Women	663	—	—	—	11.20%	8.40%	—
van Liere et al. [47]	2014	Netherlands	STD clinic	2010–2012	Women	1,321	1.30%	0.90%	2.30%	5.40%	4.80%	1.40%
Bazan et al. [23]	2015	Ohio (US)	STD clinic	2012-2013	Women	331	7.00%	6.00%	—	13.00%	13.00%	—
Danby et al. [25]	2016	Pennsylvania (US)	STD clinic	2014-2015	Women	175	2.90%	2.30%	2.30%	10.30%	11.40%	1.70%
Dukers-Muijrers et al. [19]	2015	Netherlands	STD clinic	2010–2013	Women	7,419	0.60%	0.70%	2.70%	10.20%	6.50%	1.40%
Garner et al. [27]	2015	United Kingdom	Sexual health clinic	2010	Women	649	0.80%	1.10%	0.60%	13.30%	6.60%	2.50%
Gratrix et al. [29]	2015	Canada	STD clinic	2012	Women	3055	—	—	—	9.40%	12.60%	—
Musil et al. [37]	2015	Australia	Sexual health clinic	2013-2014	Women	56	—	—	—	77.00%	57.00%	—
Trebach et al. [44]	2015	Maryland (US)	STD clinic	2011–2013	Women	4,402	2.80%	3.00%	2.10%	10.00%	8.60%	2.60%
van Liere et al. [48]	2015	Netherlands	STD clinic	2011-2012	Women	11,113	—	0.90%	—	—	9.50%	—
van Liere et al. [45]	2013	Netherlands	STD clinic	2010-2011	Women (“swingers”)	461	—	1.08%	—	—	6.72%	
Ding and Challenor [50]	2014	United Kingdom	STD clinic	2012-2013	Women	97	—	—	—	100%	77.3%	—
Cosentino et al. [51]	2012	Pennsylvania (US)	HIV clinic	2009-2010	Women	272	—	2.6%	—	—	7.7%	—
Bachmann et al. [52]	2010	US	STD clinic, HIV clinic	2003–2007	Women	99	20.30%	23.10–54.30%	8.20%	27.40%	5.60–19.20%	1.90%

*Note*. STD: sexually transmitted diseases; US: United States.

**Table 2:** Characteristics of studies reporting extragenital infections due to *N. gonorrhoeae *and *C. trachomatis* among men who have sex with men.

Author	Study year	Location	Setting	Time period	Risk	Sample	Gonorrhea			Chlamydia		
							Urogenital	Rectal	Pharyngeal	Urogenital	Rectal	Pharyngeal
McMillan et al. [73]	1981	United Kingdom	STD clinic	1981	MSM	150	—	—	—	6.70%	4.00%	1.30%
Rompalo et al. [83]	1986	Washington (US)	STD clinic	1983	MSM	1,429	—	8.00%	—	—	5.00%	—
Ostergaard et al. [38]	1997	Denmark	STD clinic	1995-1996	MSM	39	—	—	—	2.60%	2.60%	0.00%
Tabet et al. [87]	1998	Washington (US)	Community	1995	MSM	578	0.00%	0.20%	0.70%	0.00%	—	—
Kim et al. [69]	2003	California (US)	STD clinic	2000	MSM (HIV+)	564	—	7.10%	—	—	—	—
Manavi et al. [72]	2004	United Kingdom	Genitourinary clinic	1999–2002	MSM	443	—	—	—	—	7.20%	—
Kent et al. [68]	2005	California (US)	STD clinic, gay men's health center	2003	MSM	6,434	6.00%	6.90%	9.20%	5.20%	7.90%	1.40%
Currie et al. [60]	2006	Australia	Sexual health clinic	2001–2003	MSM	314	6.8% (Did not specify site)			9.6% (did not specify site)		
Hocking and Fairley [65]	2006	Australia	STD clinic	2002-2003	MSM	1,248	—	—	—	5.10%	6.20%	1.30%
Morris et al. [76]	2006	California (US)	Community	2001–2003	MSM	2,475	—	—	5.50%	—	—	—
Benn et al. [58]	2007	United Kingdom	Genitourinary clinic	1999–2001	MSM	613	7.20%	7.30%	7.30%	4.30%	6.50%	1.20%
Alexander et al. [54]	2008	United Kingdom	Genitourinary clinic	2005–2007	MSM	272	—	14.90%	6.50%	—	13.60%	—
Gunn et al. [64]	2008	California (US)	STD clinic	1997–2003	MSM	7,333	10.80%	9.80%	4.00%	—	—	—
Lister et al. [71]	2008	Australia	Sexual health clinic	2002-2003	MSM	366	—	5.00%	—	—	7.00%	—
Mimiaga et al. [74]	2008	Massachusetts (US)	Gay men's health center	2007	MSM	114	1.00%	1.70%	0.00%	2.60%	6.10%	—
Rieg et al. [82]	2008	California (US)	HIV clinic	2004–2006	MSM (HIV+)	212	1.50%	4.30%	3.30%	1.50%	2.90%	1.40%
Templeton et al. [89]	2008	Australia	Community	2003	MSM	1,427	—	—	—	—	—	1.10%
Annan et al. [55]	2009	United Kingdom	HIV/genitourinary clinic	2005-2006	MSM	3,076	—	4.10%	1.30%	5.40%	8.20%	—
Baker et al. [56]	2009	Washington DC (US)	Outpatient clinic	2006-2007	MSM	147	0.00%	1.40%	2.80%	0.00%	2.10%	0.70%
Dang et al. [61]	2009	Switzerland	HIV clinic	2007-2008	MSM (HIV+)	147	—	—	—	—	10.90%	—
Klausner et al. [70]	2009	US (5 cities)	Outpatient clinic	2007-2008	MSM	3,410/14,189	—	5.40%	5.30%	—	8.90%	1.60%
Mimiaga et al. [75]	2009	Massachusetts (US)	Gay men's health center	2003-2004	MSM	21,927	13.40%	8.80%	1.90%	5.70%	—	—
Ota et al. [77]	2009	Canada	Gay men's health center	2006–2008	MSM	248	—	11.70%	8.10%	—	7.70%	2.00%
van der Helm [49]	2009	Netherlands	STD clinic	2006-2007	MSM	1,458	—	7.00%	—	—	11.00%	—
Templeton et al. [90]	2010	Australia	Community	2003	MSM	1,427	—	—	0.60%	—	—	—
Marcus et al. [172]	2011	California (US)	STD clinic	2008-2009	MSM	3,398	0.40%	3.60%	5.00%	2.30%	7.80%	1.90%
Peters et al. [39]	2011	Netherlands	STD clinic	2007-2008	MSM	1,455	2.80%	6.00%	4.20%	4.00%	8.20%	1.50%
Soni and White [86]	2011	United Kingdom	HIV clinic	2009-2010	MSM (HIV+)	634	1.30%	4.20%	3.90%	2.60%	9.80%	1.70%
Vodstrcil et al. [93]	2011	Australia	Sexual health clinic	2002–2009	MSM	8,328/7,133	—	3.10%	1.80%	3.70%	5.40%	—
Koedijk et al. [22]	2012	Netherlands	STD clinic	2006–2010	MSM	69,506	3.40%	5.50%	3.90%	4.30%	10.10%	1.70%
Mayer et al. [36]	2012	US (4 cities)	HIV clinic	2004–2006	MSM (HIV+)	365	0.00%	2.00%	3.00%	2.00%	7.00%	1.00%
Park et al. [78]	2012	California (US)	HIV/STD clinic, gay men's health center	2010	MSM	12,454	—	—	5.80%	—	—	1.70%
Pinsky et al. [81]	2012	New York (US)	University health center	2007–2010	MSM	200	—	0.50%	3.50%	—	3.00%	0.50%
Diaz et al. [26]	2013	Spain	STD clinic	2006–2010	MSM	1,320	58.30%	21.10%	5.20%	—	—	—
Jiménez et al. [66]	2013	Spain	HIV clinic	2011-2012	MSM (HIV+)	264	—	—	9.50%	—	—	—
Sexton et al. [85]	2013	Washington DC (US)	Primary care clinic, HIV/STD clinic	2009–2011	MSM	374	—	8.00%	9.30%	—	12.70%	1.30%
Turner et al. [92]	2013	Ohio (US)	STD clinic	2010-2011	MSM	125	—	24.00%	—	—	23.00%	—
Barbee et al. [57]	2014	Washington (US)	STD clinic	2011	MSM	3,034	5.50%	9.70%	6.50%	4.40%	11.90%	2.30%
Dudareva-Vizule et al. [62]	2014	Germany	STD clinic	2009-2010	MSM	2,247	1.90%	4.60%	5.50%	3.40%	8.00%	1.50%
Gratrix et al. [63]	2014	Canada	STD clinic	2012	MSM	972	2.40%	5.90%	—	4.30%	14.10%	—
Keaveney et al. [67]	2014	Ireland	HIV clinic	2012	MSM (HIV+)	121	0.00%	4.10%	3.30%	1.70%	6.60%	0.80%
Patton et al. [79]	2014	US (42 sites)	STD clinic	2011-2012	MSM	21,994	11.10%	10.20%	7.90%	8.40%	14.10%	2.90%
Sanders et al. [84]	2014	Kenya	Medical clinic	2011	MSM	244	1.60%	5.70%	—	6.10%	8.10%	—
van Liere et al. [47]	2014	Netherlands	STD clinic	2010–2012	MSM	2,436	1.50%	3.70%	3.40%	3.30%	7.90%	1.10%
Chow et al. [59]	2015	Australia	Sexual health clinic	2007–2013	MSM	12,873	2.30%	2.90%	1.70%	3.00%	5.60%	—
Danby et al. [25]	2016	Pennsylvania (US)	STD clinic	2014-2015	MSM	224	5.40%	11.60%	16.50%	4.50%	17.40%	2.20%
Dukers-Muijrers et al. [19]	2015	Netherlands	STD clinic	2010–2013	MSM	2,349	1.40%	4.00%	3.40%	3.20%	7.30%	0.70%
Garner et al. [27]	2015	United Kingdom	Sexual health clinic	2010	MSM	365	4.70%	9.00%	5.20%	5.30%	6.50%	2.20%
Taylor et al. [88]	2015	Arizona (US)	HIV clinic	2011–2013	MSM	1,591	—	19.60%	—	—	18.60%	—
Tongtoyai et al. [91]	2015	Thailand	Medical clinic	2006–2010	MSM	1,744	1.80%	6.10%	0.50%	4.50%	9.50%	3.60%
Trebach et al. [44]	2015	Maryland (US)	STD clinic	2011–2013	MSM	769	8.90%	17.90%	11.00%	1.50%	14.30%	2.50%
Van Liere et al. [167]	2015	Netherlands	STD clinic	2011-2012	MSM	9,549	—	4.20%	—	—	9.80%	—
van Liere et al. [45]	2013	Netherlands	STD clinic	2010-2011	MSM	926	—	3.46%	—	—	7.88%	—
Bachmann et al. [52]	2010	US	STD clinic, HIV clinic	2003–2007	MSM	297	5.10%	7.90%	8.30%	2.00%	10.30%	1.70%

*Note*. STD: sexually transmitted diseases; US: United States; MSM: men who have sex with men.

**Table 3:** Characteristics of studies reporting extragenital infections due to *N. gonorrhoeae *and *C. trachomatis* among men who have sex with women and populations with mixed behaviors.

Author	Study year	Location	Setting	Time period	Risk	Sample	Gonorrhea			Chlamydia		
							Urogenital	Rectal	Pharyngeal	Urogenital	Rectal	Pharyngeal
Jones et al. [33]	1985	Indiana (US)	STD clinic	1985	MSW	706	—	—	—	21.00%	—	3.70%
Ostergaard et al. [38]	1997	Denmark	STD clinic	1995-1996	MSW	169	—	—	—	0.60%	0.00%	0.00%
Mayer et al. [36]	2012	US (4 cities)	HIV clinic	2004–2006	MSW (HIV+)	73	1.00%	0.00%	1.00%	0.00%	0.00%	1.00%
Wada et al. [173]	2012	Japan	Urology clinic	2007-2008	MSW	42	47.60%	—	11.90%	26.20%	—	2.40%
Diaz et al. [26]	2013	Spain	STD clinic	2006–2010	MSW	747	92.90%	0.00%	0.90%	—	—	—
Dukers-Muijrers et al. [19]	2015	Netherlands	STD clinic	2010–2013	MSW	5,007	0.60%	0.40%	2.20%	11.70%	0.90%	0.20%
Garner et al. [27]	2015	United Kingdom	Sexual health clinic	2010	MSW	553	0.90%	—	0.40%	12.40%	—	0.70%
Trebach et al. [44]	2015	Maryland (US)	STD clinic	2011–2013	MSW	5,218	4.30%	5.70%	2.50%	2.30%	9.10%	1.60%
van Liere et al. [45]	2013	Netherlands	STD clinic	2010–2012	MSW (“swingers”)	303	—	0.33%	—	—	1.32%	—

Ivens et al. [103]	2007	United Kingdom	Genitourinary clinic	2003–2005	Mixed	1187	—	4.70%	—	—	8.50%	—
Tipple et al. [107]	2010	United Kingdom	Sexual health clinic	2006-2007	Mixed	2,406	—	—	—	—	—	1.90%
Chan et al. [100]	2012	Rhode Island (US)	Hospital system	2011-2012	Mixed	178/21,201	0.90%	5.30%	3.40%	5.70%	11.80%	1.70%
Rodriguez-Hart et al. [106]	2012	US	Primary care/adult film	2010	Mixed	168	2.40%	2.40%	1.20%	18.50%	11.30%	22.00%
Dimech et al. [102]	2014	Australia	Surveillance system	2008–2010	Men (mixed)	177,557	—	—	—	12.2–17.4%	5.20%	1.30%
Jenkins et al. [32]	2014	Illinois (US)	Emergency room	2012-2013	Men (mixed)	192	4.70%	—	2.10%	4.70%	—	1.00%
Oda et al. [104]	2014	Japan	Otorhinolaryngology clinic	2012	Mixed	225	—	—	2.20%	—	—	0.90%
Patterson et al. [105]	2014	US	Military	2013-2014	Mixed (HIV+)	316	0.80%	4.30%	15.50%	1.00%	6.90%	6.50%
den Heijer et al. [101]	2016	Netherlands	STD clinic, gynecology, primary care clinic	2006–2010	Mixed	246/22,029	—	—	—	8.20%	10.10%	1.60%

*Note*. STD: sexually transmitted diseases; US: United States; MSW: men who have sex with women.
